# The development of co-designed parent-supported cognitive behaviour therapy for perfectionism in adolescents with eating disorders: initial feasibility and acceptability

**DOI:** 10.1186/s40337-023-00860-6

**Published:** 2023-08-17

**Authors:** Sarah J. Egan, Jamie Neal, Sarah Ure, Thomas Callaghan, Pheobe Ho, Roz Shafran, Tracey D. Wade

**Affiliations:** 1 enAble Institute and School of Population Health, Faculty of Health Sciences, Curtin University, GPO Box U1987, Perth, WA 6845 Australia; 2 Discipline of Psychology, School of Population Health, Curtin University, Perth, Australia; 3 Eating Disorders Program, Perth Children’s Hospital, Perth, Australia; 4 Eating Disorders Program, Centre for Clinical Interventions, Perth, Australia; 5 Great Ormond Street Institute of Child Health, University College London, London, UK; 6 Flinders Institute for Mental Health and Wellbeing, College of Education, Psychology and Social Work, Flinders University, Adelaide, Australia

**Keywords:** Eating disorder, Perfectionism, Co-design, Parent-supported, Intervention, Adolescent

## Abstract

**Background:**

Perfectionism is significantly associated with symptoms of eating disorders in adolescents. Studies to date have not examined parent-supported CBT for perfectionism in eating disorders (CBT-P-ED). We co-designed the treatment and conducted a feasibility trial.

**Methods:**

Eight parents of adolescents with eating disorders (*M* age = 48.75 years, 100% female) engaged in three co-design workshops to create a parent-supported CBT-P-ED self-help intervention. A further 10 parents (*M* age 41.8 years, 50% female) and their adolescent offspring (*n* = 10, *M* age 15.4 years, 60% female, 50% with self-reported diagnosis of anorexia nervosa) participated in a feasibility trial and provided feedback on the intervention.

**Results:**

The parents who engaged in the co-design workshops suggested several areas to optimise the perfectionism intervention, including using plain language, the impact of parental perfectionism, how to engage with their adolescent in treatment and the importance of increasing eating disorder specific material. Feedback from the feasibility trial suggested that the intervention was acceptable and feasible with 100% of parents and adolescents saying it was useful, and no attrition.

**Conclusions:**

Parent-supported CBT-P-ED appears to be feasible. Future research is now required in a randomised controlled trial.

## Abstract (plain language summary)

It has been repeatedly demonstrated that perfectionism can contribute to a variety of health issues. Perfectionism is a process centered on intense self-pressure to meet high standards, which has a significant impact on how an individual views themselves. Perfectionism is more prevalent in individuals with eating disorders than in those without it. There is a strong link between perfectionism and the onset of eating disorders, as well as an increase in disordered eating symptoms and a lower rate of recovery. There is currently no treatment available that addresses perfectionism in both parents and young people. The aim of the project was to work with parents and carers to co-design a parent-supported self-help program for young people that addresses perfectionism in eating disorders. We conducted three co-design workshops, followed by a four-week feasibility study to determine suitability and feedback on the intervention. The goal was to develop an intervention which can offer caregivers and young people easily available support that may aid in their understanding of perfectionism. It is hoped that by addressing perfectionism in young people with eating disorders, this approach will help them recover and thrive.

## Introduction

Perfectionism has a strong association with eating disorder symptoms [1, 2]. Perfectionism is consistently related to eating disorder symptoms in adolescents diagnosed with eating disorders [3–5]. Perfectionism has been demonstrated to predict the development of eating disorder symptoms in adolescents one year [6, 7] and four years later [8]. Adolescents with eating disorders who have higher levels of perfectionism are less likely to experience remission, and more likely to be readmitted to hospital [4].

Cognitive-Behaviour Therapy for perfectionism (CBT-P [9, 10]) is the leading evidence-based treatment for perfectionism, evaluated in over 15 randomised controlled trials (RCTs) [11]. Clinical perfectionism refers to self-worth overly dependent on achieving high standards despite adverse consequences, and the cognitive-behavioural model of clinical perfectionism [12] is used to guide CBT-P. In recent meta-analyses, compared to waitlist control, CBT-P demonstrated large effect size reductions in perfectionism and medium reductions in eating disorder symptoms over treatment [13, 14]. Perfectionism has been argued to be a transdiagnostic process [15], which is supported by a meta-analysis indicating that it is significantly associated with symptoms of eating disorders, as well as anxiety and depression [1]. CBT-P has also been demonstrated to have a transdiagnostic impact with medium effect size reductions in depression, and small-medium reductions in anxiety [13, 14].

Numerous studies in adults have demonstrated that CBT-P delivered on the internet as both guided and unguided treatment results in significant reductions in perfectionism, and symptoms of eating disorders, anxiety and depression [16–23], maintained at up to a 12-month follow-up [24]. A traditional self-help book format of CBT-P is also efficacious [25, 26], with eating disorder and associated symptoms of anxiety and depression shown to decrease in individuals with bulimia nervosa [27]. Meta-analyses have indicated no significant differences between face-to-face and internet delivered CBT-P [13, 21].

CBT-P could be enhanced through co-design [11], and one study has recently co-designed internet delivered CBT-P (ICBT-P) with adolescents [28]. The adolescent co-designed ICBT-P intervention was based on a revision of a previously evaluated website in a study with female adolescents aged 14–19 years [21]. Unguided ICBT-P showed large effects as a selective eating disorder prevention program compared to waitlist control, maintained at 6-month follow-up. [21]. Results also demonstrated transdiagnostic impacts on anxiety and depression, also maintained at follow-up [21]. Further, ICBT-P was superior to an active treatment comparison in preventing the onset of eating disorder and depressive symptoms.

While a previous study has shown that self-help CBT-P can reduce and prevent eating disorder symptoms in adolescents [21], no studies have examined if a specific eating disorder focused parent-supported CBT-P is helpful for perfectionism for adolescents (CBT-P-ED). Eating disorder treatment may be enhanced with family or carer support as recommended in the Australian and New Zealand Academy for Eating Disorders (ANZAED) guidelines that suggest treatment should attempt family involvement, no matter the age of the child [29]. Parent-supported CBT-P-ED may be helpful as an adjunctive treatment to enhance existing evidence-based treatments through providing strategies to decrease perfectionism and eating disorder symptoms in adolescents and improving understanding for parents/carers of the impact of perfectionism in the maintenance of eating disorder symptoms. Potentially parents may also help their adolescent to engage in the intervention strategies and keep them motivated to continue. Parent-led CBT for anxiety disorders is effective in younger children aged 5–12 years [30]. Importantly, perfectionism is a transdiagnostic process, and there is an overlap between anxiety, perfectionism and eating disorders [15]. Hence, it is hypothesised that parent-supported CBT-P-ED in adolescents may have important advantages in engagement and retention in treatment. Unlike parent-led CBT for anxiety which has been investigated in younger children [30], we will focus this parent-supported treatment on adolescents in the current study. While treatments for children are parent-led, we use the term parent-supported due to the treatment being flexible with adolescents having a choice over how parents can be involved, without needing to lead the treatment if the adolescent does not wish them to.

The first step in creating a parent-supported version of CBT-P-ED should be a focus on co-design of the intervention with parents and carers following the Australian National Health and Medical Research Council guidelines on co-design [31] before an evaluation of the efficacy of the intervention over a larger time frame and scale. Evidence suggests an environmental and genetic link between parent and child perfectionism in eating disorders [32]. Therefore, a focus on parents understanding and working through strategies to overcome perfectionism in their adolescent and reflecting on their own perfectionism, if present, may be beneficial.

The two aims of this study were to: (1) engage in a co-production process to produce a parent-supported CBT for perfectionism treatment for adolescents with eating disorders, and (2) trial initial feasibility and acceptability of the intervention. Given the exploratory nature and small sample involved in this initial intervention development and feasibility, no specific hypotheses were made regarding the outcome of the co-designed intervention.

## Methods

### Participants

#### Co-design phase

There were 17 parents who expressed interest in response to a social media advertisement asking for parents of adolescents with an eating disorder to participate in workshops to co-design a treatment for perfectionism. Of the 17 parents who expressed interest, 9 returned a signed consent form. One parent who completed a consent form did not further engage. The final group who engaged in the co-design workshops comprised 8 parents of adolescents with eating disorders (age range 35–65 years, *M* = 48.75 years, SD = 5.99, 100% female). Each parent was from a unique family, no parents were coparenting the same adolescent. Most parents (75%) reported that their adolescent had a current diagnosis of an eating disorder, while 25% reported that their adolescent was in remission. The most common diagnoses in adolescents that were reported by parents were anorexia nervosa (87.5%), followed by bulimia nervosa (12.5%). Some parents could not attend all online workshops and provided alternative means of feedback on the intervention materials with written feedback via email.

#### Feasibility trial

There were 18 parents who expressed interest in social media advertising for the feasibility trial. The social media advertisement was shared through parent support networks for eating disorders in Australia and the United Kingdom (UK). Of the 18 who expressed interest, 10 families completed both the parent and adolescent consent forms and were then screened for the study for suicide risk on the Columbia Suicide Severity Rating Scale [33]. All 10 parent and adolescent dyads were deemed to not be at high suicide risk and were entered into the study. The 10 parents (age range 35 to 55,* M* = 41.8 years, SD = 8.11, 5 females and 5 males) in the pilot trial were residing in Australia (*N* = 8) and the UK (*N* = 2). The 10 adolescents were also residing in Australia and the UK (age range 14–17, *M* = 15.4 years, SD = 0.97, 6 females and 4 males). The self-reported eating disorder diagnoses for the adolescents comprised 50% anorexia nervosa, 20% bulimia nervosa, 10% Binge Eating Disorder, and 20% not reported. The intervention was completed via a PDF document delivered online via email with no guidance from a therapist.

### Measures

All measures reported were administered in the feasibility trial to both parents and adolescents.

#### Perfectionism

The Clinical Perfectionism Questionnaire (CPQ) [34] was used to measure perfectionism, comprised of self-worth being based on striving to achieve demanding standards despite negative effects. The scale consists of 12 items, with items 2 and 8 being reverse scored. The CPQ has good internal consistency, convergent and predictive validity in community and eating disorder samples [35, 36], and good reliability and validity in adolescents [37].

#### Eating disorder symptoms

The 22-item global score of the Eating Disorder Examination Questionnaire (EDE-Q) [38] is a measure of eating disorder symptoms over the past month, consisting of four factors: Dietary Restraint, Eating Concern, Shape Concern, and Weight Concern. The EDE-Q demonstrates good internal consistency, test–retest reliability, and validity [39, 40].

#### Depression

The Patient Health Questionnaire (PHQ-9) [41] was used to measure depressive symptoms. The 9 items are answered on a four-point scale: *Not at all* (0) to *Nearly every day* (4). Higher scores indicate greater depressive symptoms. Scores of 0–4 indicate minimal depression, 5–9 mild depression, 10–14 moderate depression, 15–19 moderately severe depression, and 20–27 severe depression. The scale has good reliability and validity in both adult [42] and adolescent [43] populations.

#### Anxiety

The Generalised Anxiety Disorder scale (GAD-7) [44] was administered to assess anxiety. The 7 items are rated on a four-point scale ranging from *Not at all* (0) to *Nearly every day* (4). Total scores range from 0 to 28. Higher scores indicate greater anxiety. Scores of 0–4 representing minimal anxiety, 5–9 mild anxiety, 10–14 moderate anxiety, and 15 and above severe anxiety. The measure has good reliability in adults [45]. There is also evidence of good reliability and validity of the scale in adolescent populations [46].

#### Adherence to treatment

Adapted from Thiels et al. [47] study of self-help for bulimia nervosa, four items using a Likert scale were utilised as a self-report measure to determine the extent that participants reported interacting with the intervention. The items measured the degree to which participants read the self-help intervention ranging from 0%, 25%, 75%, 100%, length of time spent reading the intervention ranging from 0 min, 1–30 min, 30–60 min, 60–120 min, 120+ minutes, usefulness of the intervention (1 = *Not useful at all* to 5 = *Extremely useful*), and how easy the intervention was to read (1 = *Strongly disagree* to 5 = *Strongly agree*).

#### Ease of use

Adapted from a qualitative study of participant experiences of internet delivered CBT-P by Rozental et al. [48], two items using a three-point Likert scale were used to measure ease of use in both information read and strategies supplied in the intervention (1 = *Difficult to use* to 3 = *Very easy to use*).

#### Intervention preferences and engagement

Three items using a five-point Likert scale were used to measure participant preferences and engagement. To measure preferences, participants indicated (1 = *Strongly disagree* to 5 = *Strongly agree*) whether they would have preferred to complete the intervention alone or would have preferred further guidance. In measuring engagement, participants indicated (1 = *Strongly disagree* to 5 = *Strongly agree*) whether parents followed up on adolescent completion of activities within the intervention.

### Procedure

#### Co-design phase

To develop the intervention, we engaged in three co-design workshops with parents of adolescents with eating disorders, which were online workshops via the platform Webex. The reason that only parents were involved in the co-design workshops is that the intervention that was used as the basis to present to parents was one which was co-designed with adolescents [28]. Hence, a unique aspect of the current project was to engage with parents to seek their views about and help to co-design a parent-supported version of this previous CBT-P intervention co-designed with adolescents [28]. The co-design workshops were co-facilitated by Jamie Neal, a lived experience expert and Sarah Egan, a clinical researcher with expertise in perfectionism and eating disorders in adolescents. Parents who engaged in the co-design workshops received debriefing and information on where to engage with mental health support if required. The 8 parents who engaged in co-design of the intervention received payment of AUD$225 each.

The goal of the co-design workshops was to engage in an adaptation of an existing CBT for perfectionism treatment [21, 28]. The aim was with the addition of new information, that parents and caregivers can better understand their adolescents’ perfectionism, its connection to eating disorders, and how to reflect on their own perfectionism. To ensure that the voices of parents of adolescents with eating disorders were heard at each stage and that the information from each session was used in developing materials, it involved an iterative process over three co-design workshops. As a result, the co-design process incorporated feedback loops from parents at each stage. The final product was a PDF booklet containing text, graphics, and worksheets. The feedback provided by parents did not contradict or replace any original material that was co-designed with adolescents, except for parents suggesting that specific weights and clothing sizes should not be included, a point of feedback not made previously by adolescents [28].

#### Feasibility trial

The 10 parent and adolescent dyads who returned consent forms, registered on the study website (www.perfectionismsupported.org), where they completed a Qualtrics link with the CSS [33] suicide screening questionnaire. All parents and adolescents scored below 4 indicating that they were not at high suicide risk and were sent a Qualtrics link to complete pre-treatment questionnaires (EDE-Q, PHQ-9, GAD-7) and asked to complete the online unguided intervention consisting of two topics per week over the next four weeks. There was no therapist input during the four-week period. At the completion of the 4 weeks, participants were sent another Qualtrics link to complete post-treatment questionnaires and a range of open-ended qualitative questions for the purpose of gaining feedback on the intervention (Table 1).

**Table 1:** Qualitative feedback questions on the intervention

1. How relevant and useful was the intervention was to your own experiences (if you have them) of symptoms of perfectionism, anxiety, depression or eating disorders? If you do not have these experiences, how useful do you think the intervention would be for other parents/carers of teenagers with eating disorders for the parents/carers perfectionism or symptoms?
2. Can you please tell me how useful the intervention was for helping your teen with perfectionism, and symptoms of eating disorders, anxiety and depression? Did you follow up each session and work through it with your child?
3. What did you like about the intervention?
4. What did you not like about the intervention?
5. Any suggestions for what you think is missing from the intervention, or what you would have liked?
6. What other areas do you think could be useful or relevant for the intervention to cover?
7. Do you have any other comments you think we should consider in what would appeal to you or others in an intervention for parents/carers to support their teenager in working through perfectionism?
8. Would you have preferred to have some guidance in using the intervention or work through it on your own as you did?
9. Would you recommend the intervention to other parents/carers?
10. Do you have any further comments you think are important for us to consider?

Parents and adolescents also completed a feedback questionnaire at post-intervention, consisting of adherence to treatment, ease of use, and intervention preferences and engagement measures. Parents and adolescents received AUD$50 each at post-treatment.

The first draft of the intervention was written by SE and based on the adolescent CBT-P treatment trialled by Shu et al. [21], which was recently updated to include feedback from co-design workshops with female adolescents [28]. The intervention consisted of a 38-page PDF document consisting of 8 topics to be completed over a 4-week period (Table 2).

**Table 2:** Intervention content

Topic	Content
1	Understanding perfectionism
2	How perfectionism is maintained
3	Self-monitoring perfectionism
4	Psychoeducation (myth busting)
5	Behavioural experiments and changing ‘all or nothing’ thinking
6	Changing thinking styles
7	Procrastination, problem solving, and pleasant event scheduling
8	Self-compassion and broadening self-esteem beyond achievement

### Data analysis

The data analysis was mixed methods. Qualitative analysis followed the six steps of thematic analysis by Braun and Clarke [49]. Qualitative analysis and coding of themes was performed manually by SE with discussion and consensus of themes with JN for the co-design qualitative data. Qualitative data for the feasibility trial was analysed and manual coding of themes was performed by SU with discussion and consensus of themes with SE. Quantitative analysis was focused on the clinical significance of changes in pre-post means compared to published norms as the small sample size precluded an examination of statistical significance.

## Results

### Co-design workshop themes

The three co-design workshops resulted in feedback from parents that was organised under four themes (see Table 3).

**Table 3:** Themes arising from intervention co-design workshops with parents

1	Language
2	Parental perfectionism
3	Engagement
4	Increasing eating disorder specific material

The first theme of feedback from parents was regarding *language*. Parents spoke in all workshops about the importance of plain language and suggested that some terms should be revised in the intervention booklet to be more understandable for adolescents. Parents also repeatedly emphasised the importance of language not being critical or seen as being demanding of their child. For example, they suggested changing the term *“overcoming perfectionism”* to *“managing perfectionism”* so that there was no implicit pressure or expectation on their adolescent that they must change. Common in this topic of feedback was the response from parents of using *“softer language”* and *“the words need to be more gentle”.* In response to this feedback, the materials were revised to reduce complex language, decrease the amount of reading material and increase graphics, and remove language that parents suggested was not appropriate. For example, several parents cautioned against the use of specific examples for eating, weight and shape, suggesting that specific clothing sizes or weights be removed from the examples in the intervention booklet. Parents said this was important because (1) young people with eating disorders will vary greatly in their weight and what they think is a perfect weight or clothes size, and (2) these examples may be unhelpful or trigger distress for some young people. We revised the intervention to remove all reference to specific numbers or sizes (for example “I must weigh 50 kg” and “I must wear a size 6 dress to look good”) in line with this suggestion.

The second theme of feedback related to ways for parents to engage and reflect on their own *parental perfectionism*. Several parents commented that they felt they were perfectionists, and it would be helpful to engage in working on their own perfectionism. In response to this feedback, we increased the focus of change in parental perfectionism, and developed instructions for use of the intervention where it was suggested that parents may like to accompany their adolescent in working on perfectionism at the same time and share their learning. For example, one parent told us;

It would be helpful to think about speaking with your child about sharing the journey of changing perfectionism together so you could look at results of each other’s worksheets and share your thoughts and journey.

Another said;

I wish I had known about this treatment and about perfectionism at the start when we were dealing with an eating disorder, it would have been very helpful for us. I have learned a lot about myself reading through the intervention.

The third theme of feedback was relating to *engagement*, specifically, how parents might engage in a parent-supported treatment for perfectionism. This involved extensive discussion in the workshops, around issues of timing, for example many parents talked about how the intervention would not have been appropriate for their adolescent when they were engaged in inpatient treatment or when they were at a very low weight. Other parents commented also that it is important to think about when to do this, for example;

I think you should suggest to parents to do this when they are not distracted, at a quiet time, to remove distractions.

As a result, we included information in the notes for use to parents at the start of the intervention suggesting considering the timing of the intervention and to not use it during an inpatient admission or with severe underweight status, as it may be difficult to engage fully in the self-guided treatment. We also developed guidelines for practical suggestions about how to reduce distractions and focus on discussing the treatment between the parent and adolescent. Another aspect of this theme related to how to support their adolescent through treatment, and being careful not to push or be critical of their adolescent. For example, one parent said;

As a parent we often want to push our child to help them, but with this you would have to share working on it together, and to know when to pull back from pushing.

The final theme of feedback was in regard to *increasing eating disorder specific material.* This included comments around parents wanting to see more examples of how perfectionism and eating disorders are specifically linked, and further explanation of how perfectionism leads to the development of an eating disorder. To respond to this suggestion, we revised the intervention material to add eating disorder specific material, including the role of perfectionism in the aetiology and maintenance of eating disorders. Consequently, the current intervention is a parent-supported CBT for perfectionism treatment in the specific context of eating disorders. The information on the role of perfectionism in eating disorders would not be relevant in treatment for a general community sample of adolescents or for other presenting problems, for example, of anxiety or depression.

### Feasibility study evaluation

#### Clinical significance

The clinical significance of changes was considered based on Jacobson and Truax’s [50] criteria. Adolescent eating disorder symptoms were reduced to ‘sub-clinical’ scores at post-intervention (see Table 4) compared to clinical at pre-intervention [51]. Additionally, a clinically significant reduction in anxiety was observed in adolescents from ‘severe’ pre-intervention to ‘moderate’ post-intervention. Parents also benefited, with a clinically significant reduction from ‘moderate’ symptoms of depression pre-intervention to ‘mild’ at post-intervention.

**Table 4:** Means and standard deviations for parents (*n* = 10) and Adolescents (*n* = 10)

	Pre-treatment (*n* = 10)		Clin range	Post-treatment (*n* = 10)		Clin range	Clin M
	*M*	*SD*		*M*	*SD*		
*Parents*							
CPQ	27.73	5.68	n/a	26.80	5.03	n/a	28.53^(1)^
EDE-Q	1.43	0.85	Sub-clinical	1.54	0.83	Sub-clinical	3.98^(2)^
PHQ-9	15.82	6.72	Moderate	13.80	5.47	Mild	15.5^(3)^
GAD-7	12.55	3.30	Moderate	11.30	5.23	Moderate	14.1^(4)^
*Adolescents*							
CPQ	28.82	5.71	n/a	28.00	5.94	n/a	26.17^(5)^
EDE-Q	3.14	0.79	Clinical	2.98	1.14	Sub-clinical	3.98^(2)^
PHQ-9	19.73	5.87	Moderate	17.20	6.11	Moderate	15.5^(3)^
GAD-7	15.27	4.69	Severe	14.50	3.84	Moderate	14.1^(4)^

n/a = not applicable; CPQ = Clinical Perfectionism Scale; EDE-Q = Eating Disorder Examination-Questionnaire; PHQ-9 = Patient Health Questionnaire; GAD-7; Generalised Anxiety Disorder Questionnaire; RCI = Reliable Change Index; Clin range = Mean score of group in terms of clinical severity; Clin mean = Mean clinical norm score taken from the following studies: (1) Egan et al. [36]; (2) Jennings and Phillips [52]; (3) Richardson et al. [53]; (4) Mossman et al. [54], (5) Shu et al. [37]

#### Feedback and use of the intervention

On average, parents and adolescents reported reading 75% of each topic in the intervention, 30% reading all 8 topics in their entirety. The intervention was brief, time spent reading topics was largely less than one hour, with 50% of parents reporting spending 30–60 min reading each topic, 20% of parents reading topics in 60–120 min, and 30% of parents taking 120 min+ to complete each topic. The time spent by adolescents was also relatively brief, 20% reported spending between 1 and 30 min on topics, 30% spending 30–60 min, 30% spending 60–120 min, and 20% spending 120 min+. All participants (100%) found the information easy to use, most (90%) parents and some (30%) adolescents finding it ‘very easy’. Additionally, all parents indicated strategies were easy to use, 80% of parents perceived strategies as ‘very easy’ to use. Fewer adolescents (40%) reported strategies were ‘very easy’ to use, 40% reported strategies were ‘easy’ to use, and 20% reported strategies were difficult to use. Parents universally reported following up on strategies completed by their adolescent in the intervention. However, 20% of adolescents disagreed with the notion their parents followed up on strategies used in the intervention.

#### Qualitative analysis of feedback on the intervention from parents and adolescents

The main themes emerging from feedback from the open-ended questions (see Table 1) that parents and adolescents answered at the end of the intervention can be seen in Table 5. Participants reported how they liked the parental guidance involved in the program, the accessibility of the program’s content, and overall, the design. Participants mentioned how they would prefer an online interactive version.

**Table 5:** Themes and sub-themes from parent and adolescent feedback from feasibility trial

1. Preferences for using the guide
Parental guidance
Unguided use versus additional support
Online interactive version
2. Positive feedback on the intervention
Accessibility and applicability of the program
Design of the program
Setting goals and staying consistent

### Theme 1

#### Preferences for using the guide

A major theme emerging from the analysis was *Preferences for Using the Guide*. All 10 parents (100%) reported that they found the *parental guidance* aspect of the program beneficial, a sub-theme of this overall theme, for example one parent said,

“It helped me keep my daughter on track.” (female, age 47 years) and another stated “With my help she was able to draw out plans to meet her goals” (male, age 35 years).

Adolescents also reported liking the parental involvement. One participant said; *“I like how my Mum helped me by also reading and going through the material”* (female, age 16 years), and another said they liked *“My mum helping me with the intervention”* (female, age 15 years).

In terms of preferences for *unguided use versus receiving additional support* in using the intervention, 7 parents (70%) commented that they preferred to use the intervention without help, and 3 parents (30%) said they would have found additional guidance beneficial. An example response was *“Working through it on my own is the best”* (male, age 35 years). In contrast, 8 adolescents (80%) said they would prefer more guidance from a professional, and 2 (20%) said they preferred to complete the program independently.

When asked for feedback on how the program could be improved, 8 parents (80%) and 9 adolescents (90%) said they had nothing they would like to change. The two parents and one adolescent who did have feedback all talked about how they would prefer an *online interactive version,* and one adolescent said topic 5 was *“way too long”.*

### Theme 2

#### Positive feedback on the intervention

Positive Feedback on the Intervention from participants was identified as a major theme. Participants overwhelmingly said that the program had been useful (100% of parents and 100% of adolescents), with 4 parents (40%) and 4 adolescents (40%) saying it was extremely useful.

In terms of what participants liked most about the program, parents and adolescents both noted liking the broad *accessibility and applicability of the program*. Participants said that the best parts of the program were that it was *“Accessible and addressed perfectionism more broadly”* (female, age 55 years), and *“It has a lot of scenarios applicable to almost everyone”* (female, age 47 years).

Participants also talked about liking the design of program. For example, they said that they found the way it was written and the procedure made it easy to use, saying *“I like how the procedure went”* (female, age 35 years), and liking the *“outline, explanation, items, design”* (male, age 35 years).

Participants broadly commented that the program was most beneficial in helping them in *setting goals and staying consistent* in engaging in an intervention. For example, one adolescent saying: *“It helped me be consistent”* (male, 15 years), and a parent reported* “[it] helps me setup more goals plans and ways to achieve them”* (female, age 35 years).

#### Plan-do-study-act cycle

To incorporate this feedback into the intervention, a Plan-Do-Study-Act (PDSA) [55, 56] cycle was used. To determine initial feasibility, acceptance and feedback, co-production using methods from implementation science [55] were used. PDSA methods [56] were used to reflect on feedback from parents and adolescents and implement changes to the intervention. Specifically, this involved responding to the feedback outlined of wanting an interactive format by changing the workbook into an interactive PDF document. The other change that was made was to reduce the content of topic 5 which an adolescent had reported was *“way too long”*, by removing content on using continua to challenge ‘all or nothing’ thinking. Apart from these two pieces of feedback, there was no further specific feedback suggested for changes to the workbook by parents or adolescents in the feasibility trial.

## Discussion

The aim of this study was to (1) co-design with parents a parent-supported CBT-P-ED for use with their adolescent with an eating disorder, and (2) test the intervention to examine initial feasibility, acceptability, and feedback in a small sample of parent-adolescent dyads. The co-designed intervention with parents received positive feedback, with most parents and adolescents saying they liked the intervention, but suggesting areas to improve which were implemented before the feasibility trial. The feasibility and acceptability were high; 100% of both parents and adolescents rated the intervention as useful and there was 0% attrition between pre- and post-treatment.

The parents who engaged in the co-design workshops suggested several areas important to change in the intervention which we implemented. The importance of language was clear, with the revisions made in the process of co-design including increasing the use of simple, plain language. This highlights the benefit of the current approach in using co-design principles. Co-design may help improve engagement and a plain language approach to psychological interventions [57], which may potentially be missed in an intervention without a co-design component. An interesting point of feedback from parents in the co-design workshops was the importance of their own perfectionism in the process of intervention. Parents volunteered without prompting in the co-design workshops that trying to change their own perfectionism is important for the intervention. These viewpoints echo quantitative literature suggesting the link between parent and child perfectionism [32]. We believe that this intervention is a unique approach which may help to address this challenge of parent-adolescent dyad perfectionism and the environmental links by providing a holistic treatment designed to reduce both parental and adolescent perfectionism in the family unit. Another interesting point of feedback from parents in the co-design workshops was about how to try and engage with their adolescent in the treatment, with most suggesting that being too ‘pushy’ would not work, and repeatedly suggesting that a flexible and ‘gentle’ approach was required. In response to this feedback we created flexible use instructions, where adolescents could choose the degree and type of input from parents, from fully involved and parent-led intervention, through to general discussions without sharing details or worksheets. Potentially this flexible approach may have helped to increase acceptability in the feasibility trial, however feedback from a larger group of parents and adolescents is required to investigate this hypothesis. Finally, parents in the co-design workshops suggested we should increase specific examples of how perfectionism is related to eating disorders. This feedback was implemented to make the intervention an eating disorder specific intervention, aimed at adolescents currently experiencing an eating disorder.

Feedback from the feasibility trial of the intervention suggested that the intervention had high acceptability and feasibility with 100% of parents and adolescents reporting they thought the intervention was useful. There was no attrition between pre and post treatment, with all participants completing 100% of pre and post measures. This contrasts with the literature on self-guided adolescent CBT-P without parent involvement, where attrition was up to 50% at follow-up [21]. The lack of attrition is notable in terms of a positive feasibility outcome. Whilst we acknowledge the sample size was small, 0% attrition was noteworthy and is suggestive of acceptability of the new intervention, and the potential value of parent input in keeping their adolescent engaged in treatment [29]. The results suggest our approach of parental involvement in treatment is worthy of a RCT to examine the efficacy of the intervention.

In terms of the results of the feedback questionnaire, there was some discord between parent and adolescent reports on the intervention, with parents overall having a slightly more positive view of the intervention than adolescents. However, acceptability still appeared to be sound in adolescents, with most reporting that they found the strategies easy to use in the intervention. On average, the use of the intervention was brief and less than one hour was spent on each topic by most parents and adolescents.

The results of the intervention in the feasibility study must be considered as preliminary given the small sample size and no conclusions can be drawn from this sample size on the efficacy of the intervention. However, the clinical significance of changes in eating disorder symptoms for adolescents is consistent with adolescent only CBT-P [21], and recent meta-analyses demonstrating the efficacy of CBT-P for eating disorder symptoms [13, 14]. Similarly, the shift in clinical range for anxiety in adolescents isis also consistent with previous research on the efficacy of CBT-P [13, 14, 21]. A unique aspect of this study was examining parent mental health, and in this small sample, there was a change in the clinical range of parents’ depressive symptoms. These initial results should be viewed tentatively given the small sample size, however, support the importance of a future RCT to examine the efficacy of the intervention for eating disorder and related symptoms in adolescents and their parents.

FBT [58] is the current gold standard recommended treatment for eating disorders in adolescents and most widely studied intervention for eating disorders in this age group [59]. However, FBT does not address perfectionism as a target of treatment and the impact of FBT on perfectionism has not been examined widely [60]. It has been suggested by authors of FBT that if perfectionism is still elevated at the end of the treatment, then specific treatment for perfectionism may be indicated [61]. There has been one case series indicating that FBT supplemented with a module focused on perfectionism was useful [62], however this requires further examination in larger trials. CBT-E [63] which incorporates a focus on perfectionism when elevated, has been argued to be an alternate treatment option to FBT with some evidence for efficacy in reducing symptoms of eating disorders in adolescents [64–67]. A key point for future research on CBT for perfectionism in adolescents is to examine attrition, and whether the addition of parent involvement helps to reduce attrition in self-guided treatments for eating disorders in young people which demonstrate efficacy [68], but often have rates of attrition of up to 50% [21]. Future research should examine whether young people feel empowered and autonomous in the current intervention by making their own decisions about how parents support them through the treatment.

A notable strength of the study involved the use of principles of lived experience co-design in designing and evaluating our intervention. This approach is aligned with Australian [31] and international guidelines [69] recognising the value of incorporating lived experience to enhance mental health service quality and delivery. This is imperative for reducing mismatch between interventions and consumer preferences and ultimately increasing intervention uptake [70, 71].

A major limitation of the study was that diagnoses of eating disorders in the adolescents were self-reported, therefore caution should be taken in interpreting the results as it is not clear whether the adolescents were diagnosed with eating disorders. Given diagnoses were self-reported and recruitment was via social media, the sample may not be representative of patients seeking treatment. Another limitation was that for the self-reported diagnoses, there was a wide range of heterogeneity in diagnoses. While recent meta-analyses have emphasised the link between perfectionism and a range of eating disorder symptoms [72–74], the strong association between perfectionism and anorexia nervosa has also been noted [75], a key reason why diagnostic heterogeneity in the sample is a limitation. The conclusions which can be drawn from this study must also be considered within the feasibility trial design. The sample size for the feasibility trial was too small to engage in an analysis of the statistical significance of changes, therefore any conclusions should be tentative and viewed as evidence for the feasibility and acceptability of the approach, rather than proof of efficacy. Further, participants were not excluded from attending other services for treatment, so any change in symptoms must be considered within the context of this confounding variable. We did not seek information from participants about the other interventions (e.g., modality) that participants were engaged in, limiting the generalisability of the results due to no information about concurrent treatments in the sample.

Further research is required to develop an interactive web-based version of the intervention**.** Participants in the feasibility trial noted that an online version of the treatment was preferred and suggested an interactive, online format. The interactive PDF document that was created in response to this feedback was a first step in this process. Although this interactive PDF can be delivered via the internet and worksheets completed online, we note that there appeared to be a preference for an interactive website, like those used in our work on CBT-P in adolescents [21, 28]. Consequently, the next step following this initial feasibility trial where the PDF was delivered via email online, should be to create an interactive web-based version of the intervention in a pilot RCT.

## Conclusions

The results of the study indicate that co-designed, parent-supported CBT-P-ED in adolescents with eating disorders is a feasible intervention with high acceptability. A noteworthy and important benefit of this current parent-supported approach was that we observed no attrition, in contrast to previous research with adolescent only perfectionism treatment. Research is now required to examine the efficacy of the intervention in an RCT to determine impact on eating disorder symptoms, and perfectionism, anxiety and depression in parents and adolescents with an eating disorder.

## Data Availability

The datasets generated and/or analysed during the current study are not publicly available due to ethical considerations, but are available from the corresponding author on reasonable request and subject to Institutional approvals.
